# Predicting survival of patients with spinal involvement in multiple myeloma using PATHFx 3.0 – a validation study of 100 patients in Germany

**DOI:** 10.1186/s12957-026-04208-7

**Published:** 2026-01-27

**Authors:** Julian Kylies, Elias Brauneck, Tobias M. Ballhause, Katja Weisel, Markus Schomacher, Malte Schroeder, Peter Obid, Leon-Gordian Leonhardt, Lennart Viezens

**Affiliations:** 1 Department of Trauma and Orthopedic Surgery, University Medical Center Hamburg-Eppendorf, Martinistraße 52, Hamburg, 20246 Germany; 2 Department of Oncology, Hematology and Bone Marrow Transplantation with Section Pneumology, Hubertus Wald University Cancer Center, University Medical Center Hamburg-Eppendorf, Hamburg, Germany

## Abstract

**Background:**

Spinal malignant lesions are a common feature in multiple myeloma (MM) and often require surgical intervention. Accurate survival prediction is critical for guiding treatment decisions in these patients. While PATHFx is a widely used, machine-learning-based prognostic tool for skeletal metastases, it has not been validated for malignant bone lesions in MM so far.

**Objective:**

To evaluate the predictive performance and clinical utility of PATHFx 3.0 in a well-characterized cohort of MM patients with spinal malignant lesions.

**Methods:**

A retrospective cohort of 100 MM patients with radiologically confirmed spinal malignant lesions treated between 2009 and 2024 at a tertiary care center was analyzed. 51 patients underwent surgery for the local treatment of spinal lesions, while 49 were treated with non-operative treatment regimes. Clinical data were entered into PATHFx 3.0 to generate survival estimates at 1, 3, 6, 12, 18, and 24 months. Model performance was assessed using Receiver Operating Curves (ROC) with Area under the Curve (AUC), Brier scores, calibration plots, and decision curve analysis (DCA), and compared to the actual survival in this cohort.

**Results:**

PATHFx achieved good discriminatory performance at all time points, with AUC values ranging from 0.72 (1 month) to 0.79 (18 months). Calibration improved with longer prediction intervals, and Brier scores ranged from 0.09 to 0.20, with best accuracy at 3 months. DCA showed net clinical benefit for all models except the 1-month estimate. The inclusion of both surgical and non-surgical patients enhanced the generalizability of results.

**Conclusion:**

PATHFx 3.0 is a reliable and clinically useful tool for survival estimation in MM patients with spinal disease. Its flexible design and acceptable predictive performance support its use in multidisciplinary treatment planning, especially where traditional scoring systems fall short.

## Introduction

Multiple Myeloma (MM) is a hematologic malignancy characterized by clonal proliferation of plasma cells, with the spine being the most common site of skeletal involvement [1, 2]. Spinal malignant lesions may lead to instability, vertebral compression fractures, and neurologic compromise, often requiring surgical intervention [3–5]. However, due to the heterogeneity in disease biology, extent of spinal lesions, neurological/performance status, and systemic treatment options, not all patients with spinal involvement require surgery, which serves solely as a local intervention to maintain spinal stability and preserve neurological function, but does not address the underlying systemic disease [6–8].

Accurate estimation of individual survival is a cornerstone of decision-making in patients with malignant spinal diseases. It informs whether the anticipated survival justifies the risks of surgery and guides the choice between operative and non-operative management [9, 10]. Traditional prognostic tools, such as the Tokuhashi or Tomita scores, are point-based systems derived from historical cohorts [11–13]. Several studies have demonstrated that these scores tend to underestimate survival in contemporary patients, as they do not account for advances in oncologic therapies or individual variability in disease trajectory [14–16].

PathFx is a probabilistic, machine-learning-based tool developed to predict survival in patients with skeletal metastases. By incorporating clinical, laboratory, and disease-specific variables, PathFx provides individualized survival estimates at 1, 3, 6, 12, 18, and 24 months [17]. The model has been validated in various cohorts with metastatic bone disease, including patients with spinal metastases, and has shown superior performance compared to traditional scoring systems [18–21].

However, the predictive value of PathFx has not yet been evaluated in a dedicated cohort of patients with malignant spinal lesions due to MM. Given the distinct biological behavior of MM, often marked by prolonged survival, responsiveness to systemic therapy, and absence of visceral lesions in most cases, compared to solid tumor metastatic disease, it remains unclear whether existing prediction models perform reliably in this setting [22, 23].

Therefore, the aim of this study was to validate the predictive performance and clinical utility of PathFx 3.0 in a well-characterized cohort of MM patients with spinal involvement, including both surgically and non-surgically treated individuals.

## Materials and methods

A retrospective cohort of 100 patients with a confirmed diagnosis of MM and radiologically verified spinal involvement was analyzed. All patients were treated at a tertiary university hospital in Germany between 2009 and 2024. Of the 100 patients included, 51 (51%) underwent surgical treatment for spinal instability or neurologic compromise, while 49 (49%) were managed with solely non-operative treatment protocols. The cohort consisted of 55% male and 45% female patients, with a mean age of 67.6 ± 9.0 years. Information on bisphosphonate/osteoprotection therapy was available for all patients. Overall, 79 patients (79%) received bisphosphonate/osteoprotection therapy at or prior to the time of spinal involvement, with comparable frequencies between the non-surgical (84%) and surgical (75%) subgroups (*p* = 0.28). Table 1 summarizes the demographic and clinical characteristics of the study population.

**Table 1:** Shows the characteristics of the patients included in the study. Abbreviations: ISS: International Staging System, ECOG: Eastern Cooperative Oncology Group, ASIA: American Spinal Injury Association, EMD: Extramedullary disease

	No Surgery	Surgery	Total
Total (*n*)	49 (49%)	51 (51%)	100
Gender			
Female	22 (22%)	23 (23%)	45 (45%)
Male	27 (27%)	28 (28%)	55 (55%)
Mean Age (years)	67.1 ± 9.1	68.2 ± 8.8	67.6 ± 9.0
Cytogenetic risk			
Low	22 (22%)	24 (24%)	46 (46%)
High	19 (19%)	25 (25%)	44 (44%)
Unknown	7 (7%)	3 (3%)	10 (10%)
IgG Type	29 (57%)	22 (43%)	51
ISS			
I	12 (12%)	10 (10%)	22 (22%)
II	14 (14%)	31 (31%)	45 (45%)
III	23 (23%)	10 (10%)	33 (33%9
ECOG score	1.0 (0.9)	1.0 (0.8)	1.0 (0.9)
ASIA score (mean)	E	D	D
Visceral metastases / EMD			
Yes	1 (2%)	4 (8%)	5 (5%)
No	48 (98%)	47 (92%)	95 (95%)
Skeletal Lesions			
Solitary	10 (24%)	5 (11%)	15 (18%)
Multiple	31 (76%)	39 (89%)	70 (82%)
Lymph Node Metastases			
Yes	1 (2%)	1 (2%)	2 (2%)
No	48 (98%)	50 (98%)	98 (98%)
Pathological Fractures			
Yes	12 (24%)	18 (35%)	30 (30%)
No	37 (76%)	33 (65%)	70 (70%)
Hemoglobin (g/dl)			
Mean (SD)	10.1 (2.6)	9.5 (3.3)	9.8 (3.0)
Bisphosponate/osteoprotection use	41 (41%)	38 (38%)	79 (79%)

Clinical and disease-related data were extracted from electronic health records, including age, sex, cytogenetic risk profile, immunoglobulin subtype, ISS stage, Eastern Cooperative Oncology Group (ECOG) performance status, American Spinal Injury Association (ASIA) score, number of skeletal lesions (solitary vs. multiple), presence of extramedullary disease (EMD)/visceral metastases, lymph node involvement, pathological fractures, and baseline hemoglobin levels. Data entry was performed in structured spreadsheets.

Survival time was measured from the date of first spinal lesion detection, as this point reflects the most clinically relevant transition to vertebral involvement, often indicating a shift in disease progression and affecting decisions regarding surgical intervention, further systemic therapy, and prognosis. This method allowed for a consistent comparison of predicted versus actual survival following the onset of spinal disease.

The PATHFx 3.0 platform, which utilizes Bayesian belief networks to estimate individualized survival probabilities at 1, 3, 6, 12, 18, and 24 months, was used to calculate the estimated survival for each individual. The Bayesian modeling approach offers probabilistic estimates derived from both input variables and prior clinical data.

Predictive performance was assessed by comparing PATHFx-generated estimates with actual survival data. Discriminatory ability was evaluated using receiver operating characteristic (ROC) curve analysis, with an AUC ≥ 0.7 considered acceptable. Model accuracy was quantified using Brier scores for each survival estimate (Table 2), where lower scores indicate better prediction accuracy. Calibration plots were generated to compare predicted and observed survival rates across all time points.

**Table 2:** Shows the Brier scores for each of the different survival time points

Estimated survival	Brier score (95% CI)
1 month	0.20 (0.18–0.22)
3 months	0.09 (0.08–0.11)
6 months	0.15 (0.10–0.18)
12 months	0.14 (0.13–0.15)
18 months	0.14 (0.12–0.15)
24 months	0.12 (0.09–0.13)

To determine the clinical utility of PATHFx in decision-making in this specific cohort, decision curve analysis (DCA) was performed for all survival estimates in patients treated with surgical intervention. DCA evaluates the net clinical benefit of using PATHFx predictions compared to treating all or none of the patients as survivors at a given time point. DCA was performed exclusively in the surgically treated subgroup. The clinical decision under evaluation, whether to proceed with operative stabilization, applies only to patients eligible for surgery. Including non-surgical patients would not reflect a comparable decision context and could bias the interpretation of net-benefit estimates.

This study was approved by the local ethics board (2024-300487-WF) and conducted following the Declaration of Helsinki. Due to the retrospective and anonymized nature of the study, the requirement for informed consent was waived. All patient data were anonymized before analysis.

## Results

**Fig. 1:**
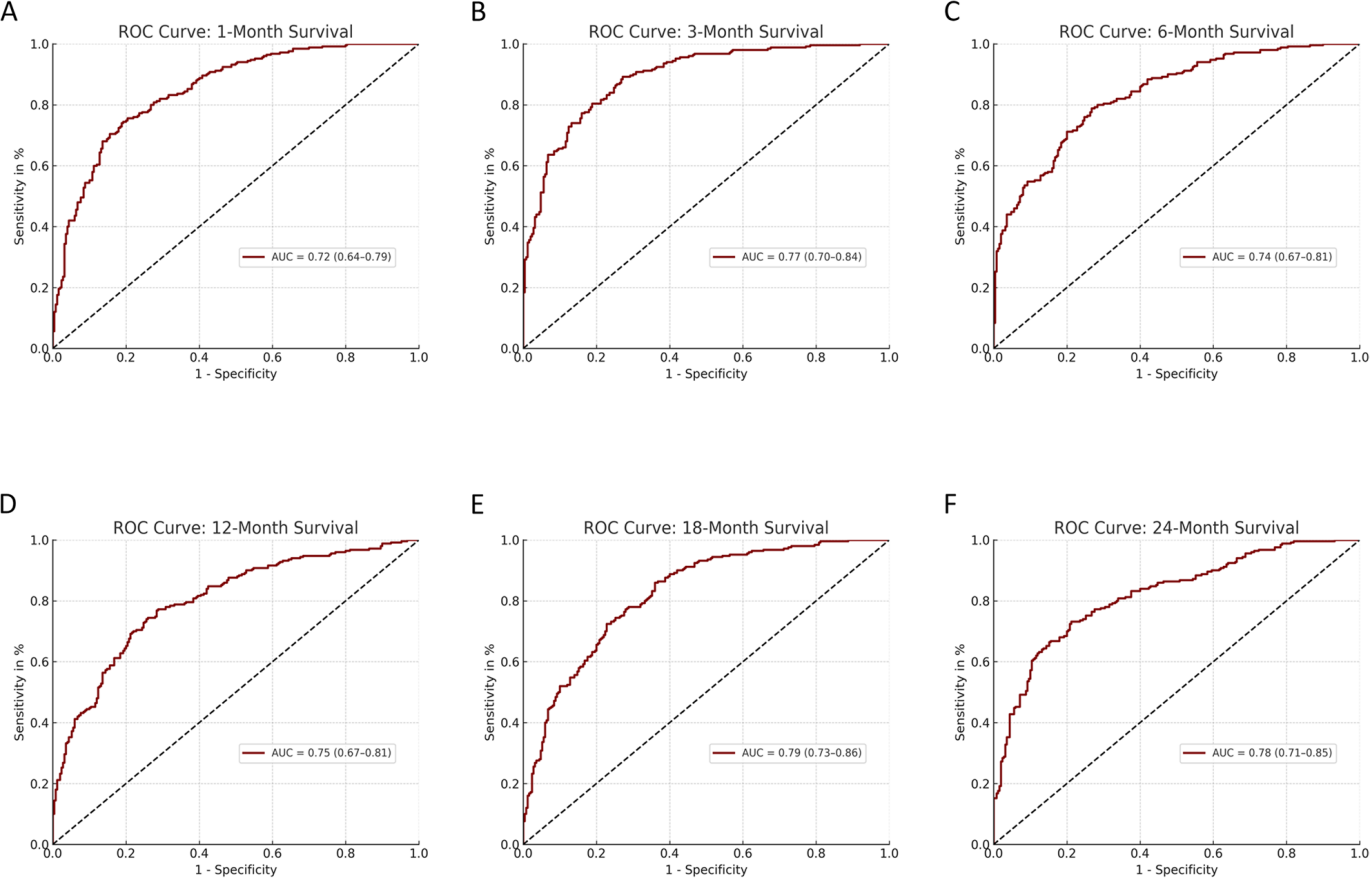
Receiver operating characteristic (ROC) curves for PATHFx 3.0 survival predictions at 1, 3, 6, 12, 18, and 24 months **A**–**F**. AUC values exceeded 0.70 at all time points, indicating good discriminatory ability

The predictive performance of PATHFx 3.0 was evaluated in a cohort of 100 MM patients with spinal involvement. The area under the receiver operating characteristic curve (AUC) for each survival model ranged from 0.72 (95% CI: 0.64–0.79) at 1 month to 0.79 (95% CI: 0.73–0.86) at 18 months, indicating good discriminatory ability across most time points (Fig. 1A–F). The AUC quantifies the model’s ability to differentiate between patients who survive and those who do not at a given time point, with a value of 0.5 indicating no discrimination and values above 0.7 generally considered acceptable. In our cohort, the highest AUCs were observed at 3 months (0.77), 18 months (0.79), and 24 months (0.78), suggesting the strongest model performance in short- to mid-term survival prediction. To quantify the statistical precision of the ROC analyses, a post-hoc precision estimate was performed by calculating the half-width of the 95% confidence interval for each AUC value. Across all time points, the mean half-width was 0.07 ± 0.02, indicating moderate precision consistent with the cohort size of 100 patients. This reflects the expected degree of uncertainty typical for exploratory validation studies.

**Fig. 2:**
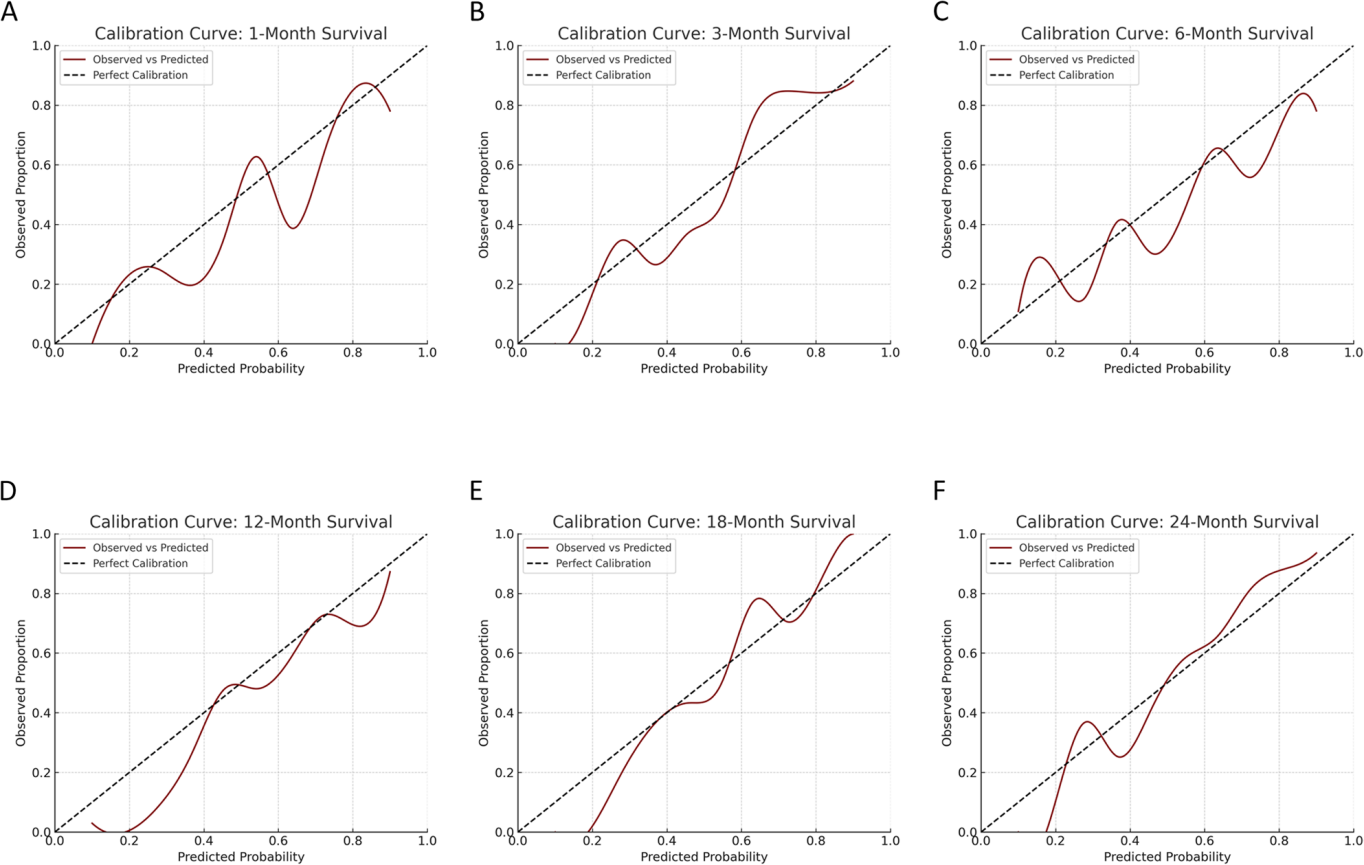
Calibration curves for PATHFx 3.0 survival predictions at 1, 3, 6, 12, 18, and 24 months **A**–**F**. The curves show the agreement between predicted and observed survival, with best calibration observed at 24 months

Calibration plots revealed that the accuracy of predicted survival probabilities improved over longer time frames. The 1-month model showed the greatest miscalibration, while maintaining adequate accuracy. In contrast, the 24-month model demonstrated the best agreement between predicted and observed outcomes (Fig. 2A–F). These calibration curves assess whether the predicted probabilities from PATHFx align with actual survival rates, thereby reflecting the model’s reliability for individual prognostication.

Prediction accuracy was further assessed using Brier scores, which measure the mean squared difference between predicted survival probabilities and actual outcomes. Lower Brier scores indicate greater predictive accuracy, with values closer to 0 representing the best model performance. Generally, Brier scores lower than 0.15 are considered to indicate adequate predictive accuracy. In our cohort, Brier scores ranged from 0.09 to 0.20 across the six time points (Table 2). The best accuracy was observed for the 3-month prediction model (0.09; 95% CI: 0.08–0.11), while the poorest performance was seen for the 1-month estimate (0.20; 95% CI: 0.18–0.22). All other models demonstrated moderate and relatively stable accuracy, with Brier scores between 0.12 and 0.15 for survival predictions at 6, 12, 18, and 24 months. To quantify the statistical precision of the Brier analyses, a post-hoc precision estimate was calculated as the half-width of each 95% confidence interval. Across all time points, the mean half-width was 0.02 ± 0.01, indicating acceptable measurement precision for most models.

**Fig. 3:**
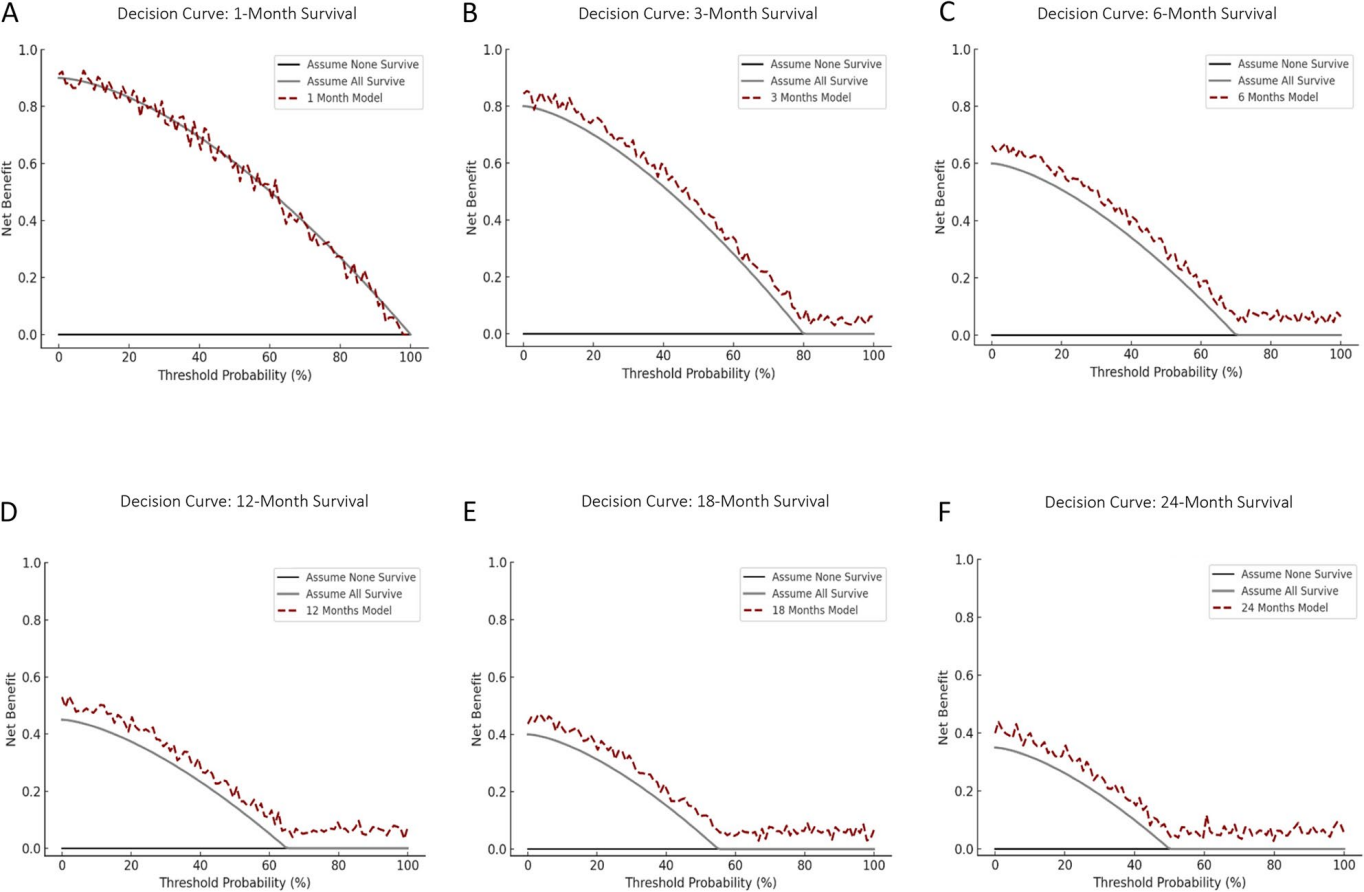
Decision curve analysis (DCA) for PATHFx 3.0 survival predictions at 1, 3, 6, 12, 18, and 24 months **A**–**F**. Net benefit was observed at all time points except for the 1-month model

DCA was used to evaluate the clinical utility of PATHFx predictions by comparing the net benefit of using the model versus treating all or none of the patients as likely survivors at each time point. Notably, only patients undergoing surgical intervention were included in the DCA analysis. The surgical subgroup comprised 28 males (55%) and 23 females (45%), with a mean age of 68.2 years (SD ± 8.8). The 1-month model provided no additional benefit over default strategies. However, for all other time points (3, 6, 12, 18, and 24 months), PATHFx predictions yielded a measurable net clinical benefit (Fig. 3A–F).

## Discussion

This study is the first to validate the predictive performance of PATHFx 3.0 in a dedicated cohort of MM patients with spinal malignant lesions. Overall, our findings demonstrate that PATHFx offers good discriminatory ability and clinically useful survival estimates in this specific patient population, supporting its applicability for decision-making in both surgical and non-surgical cases.

In the ROC curve analysis, the AUC values for all time points exceeded the commonly accepted threshold of 0.70, indicating good model discrimination across the entire time frame. Particularly strong performance was observed for the 3-, 18-, and 24-month estimates. The model’s calibration improved progressively with longer prediction intervals, and Brier scores were within the range considered good (≤ 0.15) for nearly all time points. These results highlight the robustness of PATHFx for predicting survival not only in the intermediate and long term but also in the early phase following spinal disease manifestation.

In line with previous external validations conducted in spinal metastases cohorts, our data confirm that PATHFx maintains reliable performance across diverse cancer types and clinical settings [17–20]. However, the distinct disease course of MM introduces unique prognostic challenges. Unlike many solid tumors, MM often lacks visceral metastases and tends to respond favorably to systemic therapies, resulting in a more heterogeneous and potentially prolonged survival course [24, 25]. In our cohort, most patients had multiple skeletal lesions (82%), but relatively few had extramedullary disease (5%), and overall functional status was moderately preserved. These characteristics reflect a typical MM disease pattern and may explain the comparatively favorable survival outcomes observed in this study.

In line with this, the therapeutic landscape of MM has evolved considerably over the study period ranging from 2009 to 2024, encompassing the introduction and widespread adoption of proteasome inhibitors, immunomodulatory agents, monoclonal antibodies, and more recently, cellular and bispecific immunotherapies [26]. These advances have markedly extended survival in many patients, particularly in relapsed or refractory settings, but also resulted in increased heterogeneity of clinical trajectories. Given that our cohort spans 2009–2024, it inevitably includes patients treated across distinct therapeutic eras. This variability may have influenced both observed survival and PATHFx calibration, particularly at longer prediction intervals, as the model does not account for treatment era or line of therapy. However, this heterogeneity also reflects real-world conditions in which clinical decision tools must operate. The robust performance of PATHFx across this evolving therapeutic landscape suggests that its probabilistic framework retains validity despite temporal changes in systemic treatment paradigms. Future iterations of survival prediction models may further benefit from incorporating treatment-related variables to enhance calibration in hematologic malignancies.

In comparison, traditional point-based scoring systems, such as the Tokuhashi or Tomita scores, were not designed for MM and have shown decreasing reliability in modern oncologic practice [12, 15]. More recently, the Skeletal Oncology Research Group (SORG) nomogram has been introduced as a spine-specific, machine learning-based model incorporating metastatic subtype and other prognostic factors [6]. While SORG offers valuable predictions, it is currently optimized for solid tumors, and its utility in hematologic malignancies such as MM remains unclear. One major advantage of PATHFx is that it is not limited to a specific type of cancer. Its flexible, open-source design allows it to include individual patient characteristics and be updated over time to reflect advances in treatment and clinical practice.

Another strength of our study is the inclusion of both surgical and non-surgical patients, which better reflects real-world decision-making in MM. Whereas most prior PATHFx studies were restricted to surgically treated cohorts, we show that the model retains clinical utility even when applied across the full spectrum of care. This broader applicability enhances the generalizability of our findings and supports the integration of PATHFx into multidisciplinary tumor boards, particularly when surgical indications are unclear or borderline.

Nonetheless, several limitations warrant consideration. First, this was a retrospective, single-center study, and findings may not be generalizable to centers with different treatment practices or MM populations. Second, although the cohort was well-characterized, the sample size of 100 patients remains modest and limits statistical precision. This is reflected by the relatively wide confidence intervals of AUC values in the ROC and calibration analyses. To address this, a post-hoc precision estimate was performed, demonstrating a mean AUC half-width of 0.07 ± 0.02, which indicates moderate precision consistent with exploratory validation studies in rare-disease cohorts. Third, DCA was restricted to surgically treated patients. This restriction was deliberate because DCA quantifies the clinical utility of predictions in a specific decision-making scenario, which in this study was the consideration of surgical intervention. Nevertheless, future studies could explore the application of DCA in more uniform non-surgical settings, such as during treatment decisions surrounding autologous stem-cell transplantation, to further assess the clinical value of PATHFx in broader MM care contexts. Finally, while PATHFx was compared descriptively to existing spine-specific prognostic tools, direct statistical comparison with models such as SORG was beyond the scope of this study. Future multicenter and prospective investigations should aim to validate and refine these findings in larger and more heterogeneous MM cohorts.

In conclusion, our study demonstrates that PATHFx 3.0 is a reliable and clinically useful tool for survival estimation in MM patients with spinal involvement. Given its robust performance, disease-agnostic architecture, and applicability to both surgical and non-surgical patients, PATHFx may facilitate personalized treatment planning and informed decision-making in this unique and increasingly prevalent patient population.

## Data Availability

Data can be made available upon request.
